# The cardiolipin-binding peptide elamipretide mitigates fragmentation of cristae networks following cardiac ischemia reperfusion in rats

**DOI:** 10.1038/s42003-020-1101-3

**Published:** 2020-07-17

**Authors:** Mitchell E. Allen, Edward Ross Pennington, Justin B. Perry, Sahil Dadoo, Marina Makrecka-Kuka, Maija Dambrova, Fatiha Moukdar, Hetal D. Patel, Xianlin Han, Grahame K. Kidd, Emily K. Benson, Tristan B. Raisch, Steven Poelzing, David A. Brown, Saame Raza Shaikh

**Affiliations:** 1 Department of Human Nutrition, Foods, and Exercise, Virginia Tech, Blacksburg, VA USA; 2 Department of Biochemistry and Molecular Biology, East Carolina University, Greenville, NC USA; 3 Department of Nutrition, Gillings School of Global Public Health and School of Medicine, University of North Carolina at Chapel Hill, Chapel Hill, NC USA; 4 Latvian Institute for Organic Synthesis Riga Latvia, Norwich, UK; 5 Department of Physiology, East Carolina University, Greenville, NC USA; 6 Barshop Institute for Longevity and Aging Studies, University of Texas Health Science Center, San Antonio, TX USA; 7 Department of Neurosciences, Cleveland Clinic, Cleveland, OH USA; 8 Renovo Neural Inc, Cleveland, OH USA; 9 Virginia Tech Faculty of Health Sciences, Roanoke, VA USA; 10 Fralin Biomedical Research Institute at Virginia Tech Carillion, Roanoke, VA USA; 11 Translational Biology, Medicine and Health, Virginia Tech, Roanoke, VA USA; 12 Department of Biomedical Engineering and Mechanics, Virginia Tech, Blacksburg, VA USA; 13 Virginia Tech Center for Drug Discovery, Blacksburg, VA USA; 14 Virginia Tech Metabolism Core Virginia Tech, Blacksburg, VA USA

**Keywords:** Diseases, Lipidomics, Lipids, Cardiology

## Abstract

Mitochondrial dysfunction contributes to cardiac pathologies. Barriers to new therapies include an incomplete understanding of underlying molecular culprits and a lack of effective mitochondria-targeted medicines. Here, we test the hypothesis that the cardiolipin-binding peptide elamipretide, a clinical-stage compound under investigation for diseases of mitochondrial dysfunction, mitigates impairments in mitochondrial structure-function observed after rat cardiac ischemia-reperfusion. Respirometry with permeabilized ventricular fibers indicates that ischemia-reperfusion induced decrements in the activity of complexes I, II, and IV are alleviated with elamipretide. Serial block face scanning electron microscopy used to create 3D reconstructions of cristae ultrastructure reveals that disease-induced fragmentation of cristae networks are improved with elamipretide. Mass spectrometry shows elamipretide did not protect against the reduction of cardiolipin concentration after ischemia-reperfusion. Finally, elamipretide improves biophysical properties of biomimetic membranes by aggregating cardiolipin. The data suggest mitochondrial structure-function are interdependent and demonstrate elamipretide targets mitochondrial membranes to sustain cristae networks and improve bioenergetic function.

## Abstract (web summary)

Allen and Pennington et al. show that the cardiolipin-binding peptide elamipretide mitigates disease-induced fragmentation of cristae networks following cardiac ischemia reperfusion in rats. This study suggests that elamipretide targets mitochondrial membranes to sustain cristae networks, improving their bioenergetic function.

## Introduction

The biophysical organization of the mitochondrial inner membrane regulates bioenergetics. Studies spanning fifty years have described the intertwined relationship between mitochondrial structure and function^1,2^, bolstered in more recent years by advances in imaging modalities^3–5^. The composition of the inner membrane is unique, comprised predominantly of phosphatidylethanolamine, phosphatidylcholine, and cardiolipin (CL). Notably, CL represents a structurally distinct anionic phospholipid enriched in the mitochondrial inner membrane^6,7^. CL is postulated to exist in microdomains (i.e., distinct membrane regions enriched in CL) that influence mitochondrial structure-function^8^. CL is found at negatively curved regions of the inner membrane, including cristae contact sites and along the inner leaflet of cristae tubules^6^. CL is essential for protein import, localization, and assembly, profoundly influencing mitochondrial dynamics, energetics, and network continuity^9,10^. Previous studies established oxidation and subsequent lowering of CL content across cardiac pathologies, including acute ischemia-reperfusion (I/R)^11,12^ and heart failure^13–15^. Aside from exogenous perfusion with CL^16^, which may only be applicable in experimental settings, there are currently no therapies that can improve mitochondrial function by targeting CL.

A number of cell permeable, mitochondria-targeting peptides have emerged over the last two decades. This class of peptides typically contain residues of alternating cationic-aromatic motifs ranging from 4–16 amino acids (reviewed in ref. ^17^). Elamipretide (formerly known as MTP-131, Bendavia, SS-31) is a cell-permeable peptide currently being investigated in several clinical trials to mitigate mitochondrial dysfunction associated with genetic- and age-related mitochondrial diseases. This peptide consists of a tetrapeptide sequence of D-arginine-dimethyltyrosine-lysine-phenylalanine. Preclinical studies spanning numerous models and laboratories have demonstrated preserved mitochondrial function and cytoprotection with this peptide (reviewed in refs. ^18–20^), although the mechanism of action has remained elusive. Previous work demonstrated that elamipretide interacted with CL^21^, yet the physiological consequences of this interaction are not fully understood. In this study, we utilized high-resolution mitochondrial respiration and simultaneous reactive oxygen species emission assays, biophysical membrane models, and mitochondrial imaging (serial block-face scanning- and transmission electron microscopy), to test the hypothesis that elamipretide would improve post-ischemic mitochondrial structure-function by aggregating mitochondrial CL molecules.

## Results

### Effects of I/R and elamipretide on mitochondrial respiration

**Fig. 1:**
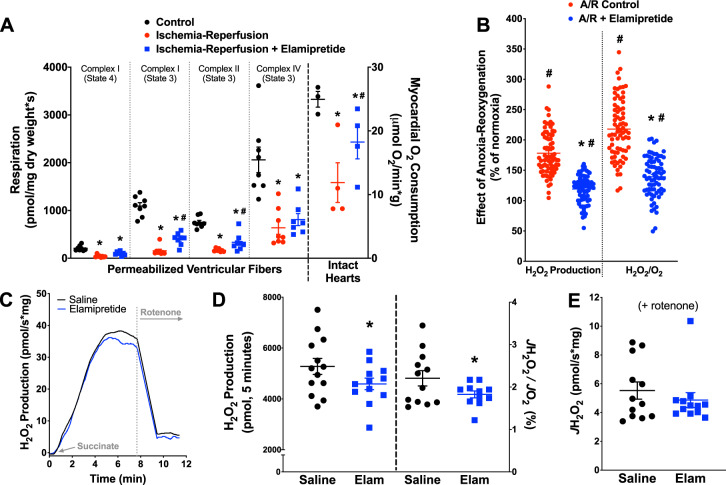
Improvement of mitochondrial function with elamipretide. **a** Decrements in mitochondrial respiration were seen across different mitochondrial complexes and substrate conditions in permeabilized fibers (*N* = 7–8 for all groups) and intact hearts (*N* = 3–4 for all groups), with elamipretide providing improved function across complexes. **b** In permeabilized ventricular fibers, elamipretide reduced H_2_O_2_ emission associated with reoxygenation (*N* = 74 for all groups). **c**–**e** Effects of elamipretide on reverse electron transport (RET) stimulated by succinate administration. Effects of elamipretide RET stimulated by succinate administration. **c** Representative trace of succinate-supported RET (*N* = 11–12 per group). **d** Elamipretide led to modest reductions in RET whether analyzed as an integrated response after succinate or steady-state (*N* = 11–12 per group). **e** Rotenone substantially reduced H_2_O_2_ emission, with no differences between saline and elamipretide groups (*N* = 12 for all groups). *P < 0.05 versus normoxic; ^#^*P* < 0.05 versus saline.

We first confirmed previous studies of myocardial uptake and mitochondrial localization using a TAMRA-conjugated elamipretide (Supplemental Fig. 1). Mitochondrial functional studies are presented in Fig. 1. In permeabilized ventricular fibers isolated after reperfusion (“Post-I/R” Fibers), respiratory control ratios (RCR; using glutamate/malate substrate) fell from 3.6 ± 0.2 in normoxic fibers to 1.9 ± 0.1 after I/R. This decrement was partially blunted with peptide treatment, with elamipretide leading to a post-I/R RCR of 2.5 ± 0.1. The substrate-uncoupler-inhibitor-titration (SUIT) protocol employed indicated decrements (average of −78% during state 3) in mitochondrial respiration across complexes I–IV after ischemia-reperfusion. Post-ischemic administration of elamipretide improved mitochondrial respiration with complex I and II substrate by an average of 56% during state 3 conditions (*P* < 0.05 compared to ischemia-reperfusion alone, Fig. 1a), and tended to improve complex IV-dependent respiration (+21%). Improved mitochondrial bioenergetics was also supported by higher myocardial oxygen consumption in the intact heart in post-ischemic hearts receiving elamipretide.

### Effects of I/R and elamipretide on H_2_O_2_ emission

A major limitation to using fibers in the above paradigm (isolating fibers from the heart after I/R) was the inability to determine whether the observed protection of mitochondrial energetics was a cause or consequence of cardioprotection. Accordingly, we devised a series of studies to determine the efficacy of elamipretide in models where energetics can be measured during the insult. Permeabilized ventricular fibers (from normoxic hearts) were placed in a high-resolution respirometry chamber and respired (saturating ADP present, “State 3”) until they consumed all of the oxygen in the chamber, thus inducing anoxia. To account for variability, each fiber preparation was normalized to its own preanoxia, normoxic value. There were observable increased levels of H_2_O_2_ at the onset of reoxygenation. Elamipretide treatment reduced fiber H_2_O_2_ emission by 33% (Fig. 1b). This effect persisted whether H_2_O_2_ rates were expressed alone or when normalized to the fiber’s simultaneous oxygen consumption rate (−36%).

We then determined if the mechanism of elamipretide involved reduction in reactive oxygen species (ROS) emission through reverse electron transfer (RET), presented in Fig. 1c–e. There was a modest but statistically significant reduction in succinate-derived RET when mitochondria were treated acutely with elamipretide. This was reflected whether the H_2_O_2_ emission was integrated over a five-minute timespan after succinate addition (−13%) (Fig. 1d) or normalized to simultaneous oxygen flux (−18%) (Fig. 1d). As expected, treatment with rotenone abolished almost all RET (rates declined from around 40 pmol/sec*mg to around 5 pmol/sec*mg). After rotenone treatment there were no differences in the rates of H_2_O_2_ production between the saline and elamipretide-treated mitochondria (Fig. 1e). We could rule out if a subtle but statistically significant decrease in H_2_O_2_ emission after rotenone would become apparent with higher N’s. Furthermore, the addition of the complex III blocker antimycin-A was not done in these studies.

These mitochondrial function studies were accompanied by studies to determine the macromolecular/supercomplex assembly of electron transport system proteins. (Supplemental Fig. 2). There was a 27% decrease in the supercomplex coupling (flux control factor) after I/R, which was improved by 10% with elamipretide (Supplemental Fig. 2D), although this did not directly correlate with changes in mitochondrial supercomplex band density (Supplemental Fig. 2A, C). There was a decrease in native complex V after I/R (−51%), which was abrogated with elamipretide (+47% versus I/R control) (Supplemental Fig. 2B, E). Although previous studies have extracted supercomplex bands from the gel and measured discernible respiration^22^, in our hands the changes in respiration when substrates were given appeared to be an artifact, as it was observed even when nonloaded acrylamide gel was placed into the respirometer.

### Effects of I/R and elamipretide on mitochondrial structure

**Fig. 2:**
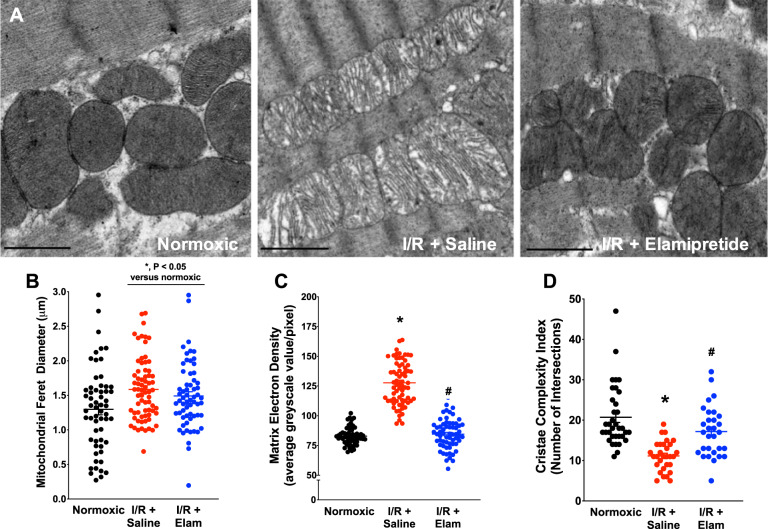
TEM data from hearts in the study. **a** Representative images from experimental groups. **b** Images were analyzed for mitochondrial Feret diameter (*N* = 59 for normoxic, *N* = 70 for I/R + saline, and *N* = 62 for I/R + Elam), **c** matrix electron density (*N* = 59 for normoxic, *N* = 70 for I/R + saline, and *N* = 62 for I/R + Elam), and **d** cristae complexity index (*N* = 35 for normoxic, *N* = 30 for I/R + saline and I/R + Elam). *P < 0.05 v normoxic; ^#^*P* < 0.05 versus I/R + Saline. Scale bar represents 2 µm.

Given the integral relationships between mitochondrial function and structure^4,23^, we employed two different electron microscopy imaging modalities to determine mitochondrial morphology. Results from transmission electron microscopy are presented in Fig. 2, with representative images in Fig. 2a and Supplemental Fig. 3. Ischemia-reperfusion induced an 18% increase in mitochondrial swelling, with I/R-saline-treated hearts displaying a greater Feret diameter when compared to normoxic hearts (Fig. 2b, *P* < 0.05 versus control). Treatment with elamipretide did not markedly influence mitochondrial swelling based on transmission electron microscopy (TEM) imaging. I/R induced a 35% decrease in mitochondrial electron density (*P* < 0.05, Fig. 2c), which was attenuated with elamipretide treatment by 34%. Sarcomeric contracture (z-band width) was prominent with ischemia-reperfusion (1.48 μM Normoxia vs. 1.18μM I/R + Saline, *P* < 0.05), and was not affected by post-ischemic elamipretide treatment (1.18 μM I/R + Saline vs. 1.14 μM I/R + Elamipretide). These sarcomere lengths were shorter than other reported values in whole fixed tissue, which may be due to prolonged perfusion with a crystalloid buffer or inconsistencies in the cutting plane of our samples^24–27^.

Mitochondrial cristae complexity index was lowered after reperfusion (−46%), and this decrease in cristae complexity was attenuated with elamipretide by 36% (Fig. 2d). Cristae width averaged 25.0 ± 1.8 nm in normoxic mitochondria (*N* = 189) and was not influenced by I/R (cristae width: 25.4 ± 0.7 nm; *N* = 178) or elamipretide treatment (26.7 ± 0.5 nm; *N* = 172).

**Fig. 3:**
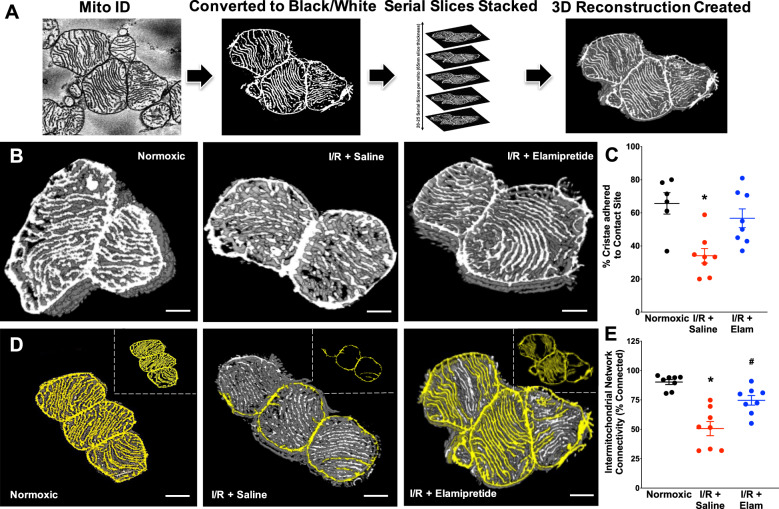
Serial block-face scanning electron microscopy images of mitochondrial ultrastructure in the experimental groups. Original SBF–SEM serial images were acquired and processed into 3D reconstructions using ImageJ (**a**). Mitochondria were then analyzed for contract site analysis (**b**) and intermitochondrial network connectivity (**d**), with quantified data in panels **c** and **e**, respectively. *N* = 6–8 for normoxic, *N* = 8 for IR + Saline/Elamipretide groups *P < 0.05 versus normoxic, ^#^*P* < 0.05 versus I/R saline. Three-dimensional reconstructions of these images are presented in the Supplemental Figures, Fig. S5. Scale bar represents 500 nm.

**Fig. 4:**
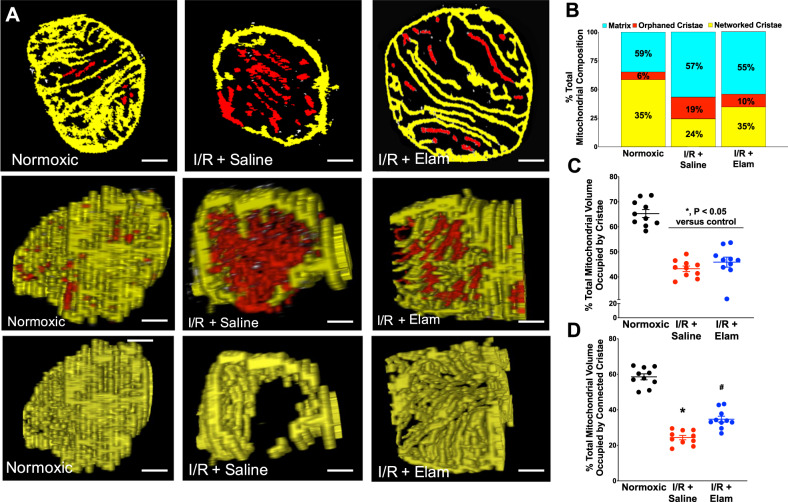
Serial block-face scanning electron microscopy (SBF–SEM) images of single mitochondrial ultrastructure in the experimental groups. Mitochondria were analyzed for cristae connectivity. **a** Top panel: individual slices from SBF–SEM imaging showing cristae networking. Middle panel: cross-section of composite stacks indicating networked (yellow) and orphaned cristae (red). Bottom panel: mitochondrial cross-section showing only networked cristae (from middle panel). Composition of total mitochondrial volume (**b**), total cristae volume (**c**), and connected cristae volume (**d**) were measured in each experimental groups. *N* = 10 for all groups. Scal bar represents 250 nm. *P < 0.05 versus normoxic; ^#^*P* < 0.05 versus I/R.

Higher resolution serial block-face scanning electron microscopy (SBF–SEM) was employed to obtain more advanced structural insight of cardiac mitochondria after I/R. These data are presented in Figs. 3 and 4 (along with reconstructed three-dimensional movies in Supplemental Movies 1–3). Under normoxic conditions, approximately 70% of cristae were physically adhered to the inner boundary membrane, termed “contact sites”. I/R injury led to a decrease (−37%) in the number of cristae contact sites (Fig. 3a, b; *P* < 0.05 versus normoxia). Post-ischemic administration of elamipretide blunted the loss of cristae adhered to contact sites (+23% versus I/R injury) (Fig. 3b; *P* < 0.05 versus I/R alone). Among adjoining mitochondria, intermitochondrial cristae network connectivity analysis indicated a substantial loss (−40%) in network connectivity between mitochondria after I/R (presented in Fig. 3c, d). Intermitochondrial cristae connectivity improved in post-ischemic hearts perfused with elamipretide by 24% (Fig. 3c, d).

Separate SBF–SEM analyses were conducted to determine the extent of intra-mitochondrial cristae network connectivity. In these studies, we attempted to map the connectivity of cristae inside a subset of mitochondria using the rationale that contiguous cristae facilitate efficient energy transfer. We termed cristae that were interconnected “networked cristae”, versus cristae that were disconnected from the network as “orphaned cristae”. From the analysis, we rendered networked cristae yellow and orphaned cristae red. These data are presented in Fig. 4 (with reconstructed three-dimensional movies in Supplemental Movies 4–6). Interestingly, in normoxic hearts 90% of all cristae appeared to be networked with one another (Figs. 4b–d and S7), 22% more matrix volume swelling, and a 22% loss of cristae volume occurred after reperfusion, which were not prevented with elamipretide (Fig. 4a–c). However, elamipretide increased the number of “connected cristae” by 10% versus cristae that were orphaned from the network (Fig. 4a, d). Furthermore, cristae that were reconstructed appeared tubular. In healthy mitochondria, cristae were often long, formed a lattice, and attached to the boundary membrane. These features are similar to the “finger-like” digitiform cristae structure as previously described^28^. Taken together, these data indicate that elamipretide did not prevent mitochondrial swelling or the loss of total cristae at reperfusion, but improved cristae contact sites with the boundary membrane and reticular connectivity among the cristae network.

### Mass spectrometry analyses of CL content and composition

**Fig. 5:**
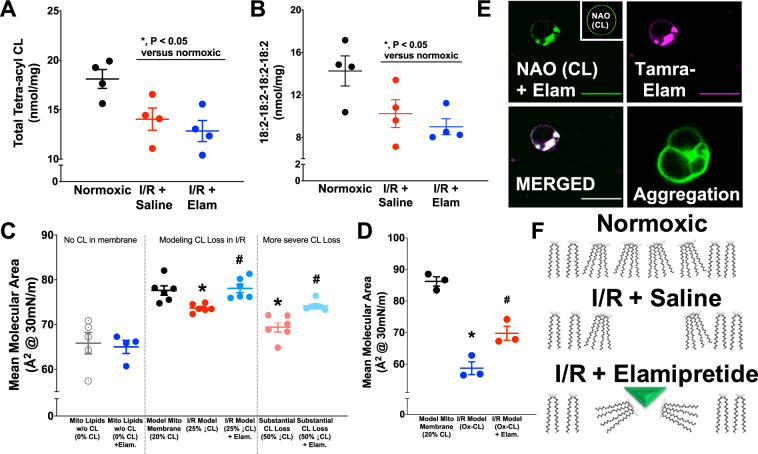
Studies of elamipretide interactions with CL. Lipidomics indicated declines in total (**a**) and tetra-linoleoyl CL (**b**) after ischemia-reperfusion. Biomimetic models of the mitochondrial inner membrane lipids presented in **c**–**f**. **d** GUV imaging of mitochondrial CL (NAO) and fluorescent TAMRA-elamipretide indicate elamipretide-mediated CL and vesicle aggregation (*N* = 3 experiments, 40–100 images per group). **c** Mean molecular area at physiological membrane pressure (30 mN/m) indicates consequences of losing CL content (*N* = 3–6 per group). Elamipretide augmented mean molecular area despite CL losses (modeled after the mass spectrometry data in panel **a**. More severe (50%) declines in total CL were also modeled. **d** Biomimetic membrane studies also determined effects of CL peroxidation and effects of elamipretide. **f** Proposed model in which elamipretide aggregates CL headgroups to increase mean molecular area. *P < 0.05 v control, #*P* < 0.05 v. I/R.

The functional and structural importance of CL led us to conduct shotgun lipidomic studies for CL content and acyl chain composition. These data are presented in Fig. 5a, b, with the 20 most abundant CL species presented in Table 1. There was a 23% decrease in the total amount of CL after ischemia-reperfusion, as well as a 28% decline in the most abundant CL species (18:2-18:2-18:2-18:2) CL. The acute administration of elamipretide at the onset of reperfusion did not abrogate a reduction in CL content, the decrease in (18:2-18:2-18:2-18:2) CL species, or alter any of the other CL species examined (Fig. 5a, b and Table 1).

**Table 1:** Myocardial CL levels after ischemia-reperfusion, expressed as a percentage of total CL per sample (data are the top 20 most prevalent CL species from mass spectrometry studies).

CL species	Control	Ischemia-reperfusion	Ischemia-reperfusion + elamipretide
18:2-18:2-18:2-18:2	74.36 ± 4.38	68.17 ± 3.47	65.58 ± 3.03
18:2-18:2-18:2 (MLCL)	4.47 ± 0.33	4.69 ± 0.63	5.78 ± 0.35
18:2-18:2-18:2-22:6	3.53 ± 0.70	3.80 ± 0.47	3.82 ± 0.62
18:2-18:2-18:2-20:3	2.91 ± 0.08	2.98 ± 0.14	2.96 ± 0.15
18:2-18:2-18:2-20:4	2.30 ± 0.27	3.12 ± 0.22	3.05 ± 0.22
18:2-18:2-18:2-22:5 or 18:1-18:2-18:2-22:6	1.63 ± 0.26	2.05 ± 0.20	1.99 ± 0.19
18:2-18:2-18:2-20:2	1.40 ± 0.13	1.56 ± 0.07	1.49 ± 0.10
18:2-18:3-18:2-18:2	1.23 ± 0.06	1.03 ± 0.07	1.06 ± 0.06
18:2-18:2-18:2-16:1	1.17 ± 0.10	1.53 ± 0.05	1.28 ± 0.03
18:1-18:2-18:2-20:2	0.37 ± 0.04	0.40 ± 0.03	0.38 ± 0.04
18:1-18:2-18:2-22:5 or 18:1-18:1-18:2-22:6	0.35 ± 0.06	0.51 ± 0.05	0.49 ± 0.04
14:0-16:1-16:1-16:1	0.29 ± 0.04	0.24 ± 0.06	0.07 ± 0.03
18:2-18:2-18:2-16:0	0.29 ± 0.02	0.36 ± 0.03	0.32 ± 0.03
18:2-18:1-18:1-16:1 or 18:2-18:2-18:1-16:0	0.17 ± 0.03	0.24 ± 0.03	0.21 ± 0.03
18:2-18:2-16:1-16:1	0.16 ± 0.01	0.22 ± 0.01	0.19 ± 0.01
18:2-18:2-18:1 (MLCL)	0.15 ± 0.04	0.22 ± 0.05	0.26 ± 0.03
16:1-18:1-18:1-18:1	0.12 ± 0.03	0.21 ± 0.03	0.17 ± 0.03
18:2-18:3-18:2-20:4 or 18:2-18:2-16:1-22:6	0.07 ± 0.02	0.10 ± 0.01	0.10 ± 0.02
18:2-18:1-20:4 (MLCL)	0.07 ± 0.01	0.11 ± 0.02	0.14 ± 0.01
18:2-18:3-18:2-16:1	0.03 ± 0.01	0.04 ± 0.01	0.04 ± 0.01

MLCL represents monolyso CL species.

### Biophysical studies of elamipretide

To better understand the biophysical interaction of elamipretide with cardiac mitochondrial membranes, we synthesized biomimetic membranes of the inner mitochondrial membrane. The model allowed us to tightly control lipid composition. Mitochondrial models were composed of biologically relevant lipids (see methods), and a CL content that represented 20% of the total lipids (consistent with content percentages seen across mammalian mitochondria^29^).

Informed by our CL lipidomic studies (Fig. 5a, b), we modeled the effects of decreased CL content after ischemia-reperfusion in biomimetic mitochondrial membranes by examining the biophysical interactions using a Langmuir trough (which provided mean molecular pressure-area isotherms of compressed lipid monolayers). A 25% reduction of CL content (“I/R model”) resulted in a 5% reduction in the mean molecular area at a physiological membrane pressure of 30 mN/m (Fig. 5c). Acute addition of elamipretide to I/R biomimetic membranes restored the mean molecular area in biomimetic monolayers with reduced CL (Fig. 5c) and oxidized CL (Fig. 5d). We also modeled severe reduction of CL content (50%, more comparable to some genetic mitochondrial diseases) and also saw a 7% improvement with elamipretide treatment versus I/R (albeit not restoring the area to the non-pathological membrane levels). Notably, elamipretide treatment of biomimetic monolayers without CL present had no discernible effect on membrane behavior (Fig. 5c, left panel).

To compliment the monolayer studies, we synthesized biomimetic mitochondrial lipid vesicles of similar composition. In the absence of peptide, CL fluorescence (assessed by nonyl acridine orange (NAO) localization) was uniform across the vesicle membrane and was accompanied by no observable aggregation of adjacent vesicles. Upon the addition of elamipretide to biomimetic vesicles, the NAO signal clustered into enriched domains. This CL clustering colocalized with fluorescent TAMRA-elamipretide (Fig. 5e), providing a complement to our imaging in intact cells (Supplemental Fig. 1). Furthermore, addition of elamipretide promoted aggregation of adjacent lipid vesicles (Fig. 5e) suggesting a potential mechanism of action (Fig. 5f). This aggregation effect was not seen in vesicles devoid of CL, and our previous work has shown that proteins that do not associate with CL do not have this effect on CL aggregation^30^.

Additionally, we serendipitously discovered that CL-containing vesicles and elamipretide eventually precipitated when in solution together (Supplemental Fig. 4). Titration studies indicated that elamipretide aggregated CL-enriched vesicles, essentially saturating the signal at a molar ratio of one peptide to two CL (Supplemental Fig. 4).

## Discussion

This study advanced our understanding of mitochondrial structure-function in hearts exposed to ischemia-reperfusion. First, we found parallel decrements in mitochondrial cristae ultrastructure and respiratory function noted across electron transport system complexes after ischemia-reperfusion. Second, we used an innovative approach to map mitochondrial network connectivity in the heart, discovering decrements among and between mitochondrial cristae networks after ischemia-reperfusion. This aspect of our study employed the application of serial block-face scanning electron microscopy (SBF–SEM) to determine and quantify changes in mitochondrial cristae structure and connectivity in cardiac pathology. Third, the studies provided new mechanistic insight into elamipretide, a clinical-stage peptide that appears to aggregate CL and improve mitochondrial membrane structure and bioenergetic function without preventing the acute, reperfusion-induced decrease in CL content. Finally, we employed the use of biomimetic membranes to directly quantify membrane-dependent effects of pathologies and putative therapeutics.

The mitochondrial network is an attractive target for adjuvant therapies^20,31^. The factors that promote bioenergetic impairments include: reactive oxygen species production that exceeds endogenous scavenging capacity, matrix calcium overload, imbalances in substrate content/composition, inner membrane uncoupling, membrane lipid oxidation/degradation, inefficient electron flux, opening of energy-dissipating channels/pores, and collapse(s) in mitochondrial membrane potential (reviewed in refs. ^8,32,33^). A number of different pharmacological approaches have investigated the aforementioned factors, with several compounds progressing to clinical trials (reviewed in ref. ^34^). Our work advanced the field by showing that mitochondrial function could be improved by targeting decrements related to CL.

**Fig. 6:**
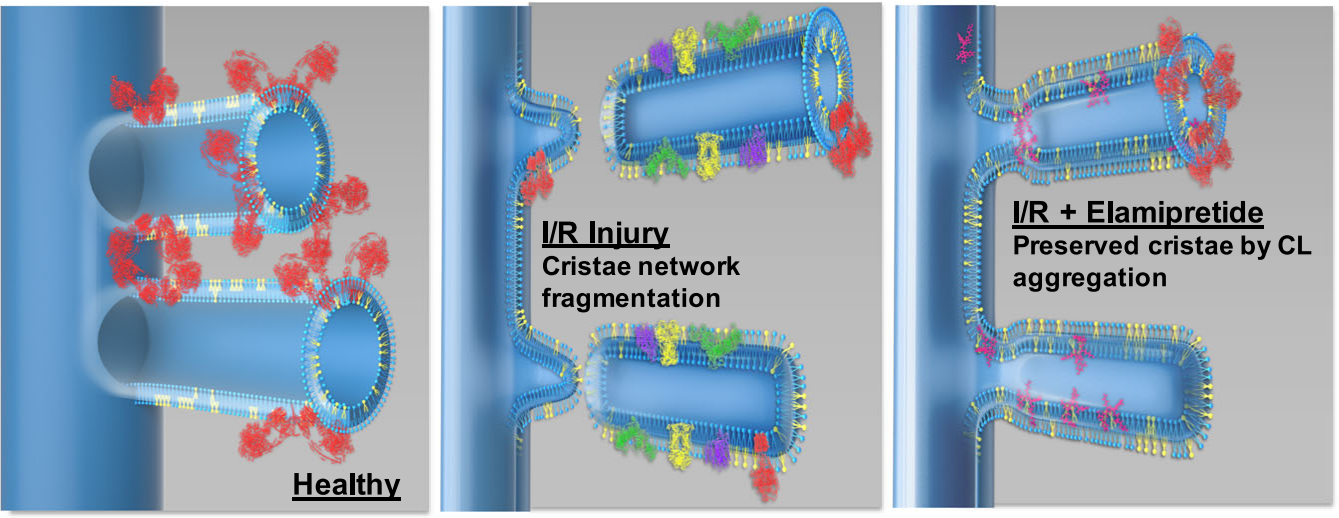
Proposed model in which elamipretide (depicted in magenta—right panel) aggregates CL (depicted in yellow) to preserve cristae ultrastructure in diseased mitochondria. Preserved cristae integrity was associated with protection of complex V (red) band density. The other electron transport chain complexes are depicted in green, yellow, and purple (middle panel). Therapeutic approaches that target CL may conserve inner mitochondrial membrane integrity, maintain cristae contact sites, sustain intra and intermitochondrial networks, and improve mitochondrial function during disease states.

CL contributes to membrane structure by imparting negative membrane curvature^35^ at cristae junctions and along the inner leaflet of cristae membranes (depicted in Fig. 6). CL also serves as a membrane anchor for mitochondrial proteins essential for the cristae assembly/morphology, including ATP synthase dimers^36–40^, OPA1^40–43^, mitoregulin^44^, and components of the mitochondrial contact site and cristae organizing system (MICOS)^10,45–49^. Although the role of proteins in influencing cristae structure cannot be understated, proton-dependent cristae formation was observed in membrane lipid vesicles devoid of any proteins^50^.

A reduction in the concentration of (18:2-18:2-18:2-18:2) CL that we observed was consistent with previous studies (refs. ^12,16,51,52^). Likewise, mitochondrial ultrastructural defects, similar to what we observed, are reported across other pathologies^23^ characterized by a decrease of CL concentration and corresponding cristae^7^. CL replacement strategies have been tested and include direct perfusion of CL to isolated rat hearts^16^ and utilization of CL-containing nanodiscs^53^. While promising, the translational relevance of these paradigms for patients remains to be demonstrated.

Our finding of multi-complex dysfunction after ischemia-reperfusion compliments previous studies noting decrements in complex activity, subunit oxidation, and augmented ROS production along the electron transport system^33,54–63^. Mitochondria-targeting peptides represent an emerging class of therapeutics that are being tested across diseases. These peptides share structural homology with endogenous mitochondria-targeting sequences, which are both amphipathic and cationic^64^. Most mitochondria-targeting peptides contain alternating cationic-aromatic amino acid motifs^17,65,66^. Mitochondria-targeted peptides appear to be lipophilic enough to cross membrane barriers, typically contain arginine (especially the D-isomer for enzymatic stability), and are postulated to hone to mitochondria based on the negative membrane potential, the presence of anionic phospholipids (such as CL), or combinations thereof.

Elamipretide is a salt-variant of the SS-31 peptide first serendipitously discovered by Szeto and Schiller^67^ in their search for opioid receptor ligands. This peptide showed protective efficacy across preclinical studies (reviewed in refs. ^8,19^). Improved post-ischemic respiratory function with elamipretide was observed across complexes, in agreement with previous studies where elamipretide improved activity or expression of several different electron transport complexes^15,68–70^. These data suggest that elamipretide’s mechanism of action does not depend on one particular protein or complex. The improved bioenergetic function across complexes was suggested by studies examining the existence of native protein complexes. Supercomplex coupling control factor^71^, a functional measure of the ‘intactness’ of respirasomes, and native complex V structure were both impaired after ischemia-reperfusion and improved with elamipretide (Supplemental Fig. 2D, E). Although the functional significance of improved complex V density was not directly tested, the mitigation of electron transport chain dysfunction observed with elamipretide suggests the peptide may lessen decrements in ATP production previously observed after I/R^72^. We did not find evidence of a robust decrease in supercomplex band density after ischemia-reperfusion. These results are consistent with other studies examining supercomplexes after acute cardiac ischemia-reperfusion^73,74^, which generally show modest effects of ischemia-reperfusion on supercomplex band density. These findings also corroborated previous work where cardioprotection was not associated with augmented supercomplex band density^75^. Given the sensitivity of native protein complexes to detergent conditions^74,76,77^ and the issue of sample bias in isolating mitochondrial supercomplexes from infarcted myocardium (i.e., isolation procedures enrich for healthy mitochondria), future studies are warranted to better understand the relevance of blue-native supercomplexes in models of cardiac pathology.

The SS-31 peptide was originally described as a “scavenger” of reactive oxygen species. While it is clear that elamipretide can reduce overall ROS levels from pathological mitochondria (refs. ^15,78^, and Fig. 1 herein), several lines of evidence suggest that it is not scavenging ROS. We previously showed that the peptide was not scavenging either superoxide or hydrogen peroxide using cell-free model systems^18^. Other groups reported that elamipretide-mediated reductions in ROS are observed in diseased tissues but not healthy tissues^68,79^, also suggesting that the peptide may be reducing pathological production of ROS and not scavenging per se. Preconditioning of the heart, which has been shown to involve small bursts of ROS that trigger adaptive responses and is typically abolished with “ROS scavengers”, was not abolished with elamipretide^80^. Our finding of reduced ROS emission in permeabilized ventricular fibers and a modest reduction in RET^81^ provides further evidence that the peptide may be reducing succinate-derived RET early in reperfusion. Interestingly, elamipretide-mediated reductions in ROS production by the electron transport chain (ETC) were not observed downstream of ubiquinone (Fig. 1), suggesting an alternative mechanism than one involving cytochrome c-mediated injury^21,82^.

We observed interactions between elamipretide and CL, confirming findings first made by Birk et al.^21,82^. Among endogenous proteins, cationic amino acid residues are known to associate with CL^44^. Results from our microscopy studies with NAO and elamipretide colocalization suggest the peptide interacts with CL, which we speculate was driven by the cationic amino acids. We acknowledge that a limitation of our work is the use of a simplified biomimetic model system that lacks proteins. Future studies will require the incorporation of differing proteins into biomimetic membranes. An additional potential limitation of our work could be the use of imaging, which could introduce artifacts during sample preparation and analyses. Therefore, future studies will need to effectively integrate imaging, biochemical, and biophysical approaches.

Acute elamipretide treatment at reperfusion did not prevent the loss in CL content observed after acute ischemia-reperfusion. While longer term administration of elamipretide (>4 weeks) normalized aberrant CL in canine models of heart failure^15^ and pigs with metabolic syndrome^70^, our data indicated that the peptide has acute activity to preserve mitochondrial structure-function even in the presence of CL deficiencies. This beneficial activity suggests that the peptide may be effective in diseases characterized by a reduction of CL concentration (such as genetic mitochondrial diseases and/ or Barth syndrome [BTHS]).

The lowering or oxidation of CL modeled after ischemia-reperfusion led to a discernible change in the membrane structure (Fig. 5). Elamipretide promoted a physical ‘aggregation’ of CL molecules where the peptide is acting like a membrane adhesion factor for the CL that is present (even if it is oxidized). As it is difficult to study mammalian mitochondria devoid of CL, our finding that elamipretide-mediated lipid aggregation is not present without CL highlights the preferential nature of this interaction.

The nature of the physical aggregation between CL and elamipretide remains to be studied in the future. One approach would be to use fluorescently labeled CL to understand its complex relationship with the peptide; however, it will be critical to demonstrate that fluorescently labeled CL shows the same biophysical properties as native CL, which is unlikely to be the case due to the presence of bulky fluorescent groups. Additional studies are needed to understand the nature of the physical interaction of CL with the peptide as a function of changes in surface pressure. Our experiments relied on 30 mN/m as this is the gold standard in the field for biological relevance; however, surface pressure in the inner mitochondrial membrane likely varies with curvature and matrix swelling. To the best of our knowledge, the surface pressure as a function of cristae curvature remains to be established but is an important area for study.

The finding of improved mitochondrial ultrastructure with elamipretide in cardiac ischemia-reperfusion injury agrees with previous work where elamipretide improved mitochondrial morphology in other diseases^70,82,83^. We specifically provide insight into mitochondrial structure using higher resolution, SBF–SEM, which is an imaging approach capable of creating high-resolution 3D images that provide quantitative data on mitochondrial cristae morphology. The technical operation is also less demanding than other imaging approaches for performing volume analyses (i.e., serial section TEM and focused ion beam SEM), and SBF–SEM analysis is within the capabilities of many labs that lack previous EM experience^84^. The relationship between changes in mitochondrial morphology and functional outcomes remains unclear as reported here and by others^85^. Therefore, there is a critical need to further understand how modest changes in morphology could be driving key changes in the biophysical properties of the inner mitochondrial membrane to control respiratory function, an area for future investigation.

The finding that cristae contact sites were disrupted in the post-ischemic heart compliment altered contact sites seen in other diseases^86,87^. Elamipretide did not prevent the loss in total cristae with reperfusion (Fig. 4c), consistent with a lack of protection against decreased CL content (Table 1 and Fig. 5a, b), as these are inter-related^50,88^. A major advancement using this technology was that elamipretide partially restored the cristae network connectivity. This may help explain the protection of ATP synthase dimers with elamipretide, as ATP synthase and cristae architecture have shown interdependent declines in other pathologies^38^. However, more studies are needed to confirm this hypothesis as we did not specifically study the interdependence of cristae shape and ATP synthase. The modest reduction in RET we observed was also associated with improved cristae ultrastructure. Accordingly, it might be hypothesized that succinate accumulation induces RET, which has been associated with mitochondrial fragmentation^89^.

If any generalizations can be made about elamipretide’s mechanism across studies, it is that this peptide appears to exert biological effects predominantly when there is an underlying pathological burden present. In these cases, it tends to ‘normalize’ mitochondrial function that is dysfunctional prior to elamipretide exposure. The ability of elamipretide to prolong PTP opening^15,18^, improve exercise capacity^79^, promote CL remodeling^15,70^, reduce apoptotic signaling^90^, stimulate state 3 respiration^21^, or improve the activity of several different ETC complexes^15,68–70^ is only observed in diseased, aged, or damaged (respiratory control ratio <2) mitochondria. There appears to be translational support for this concept, as a recent clinical trial with elamipretide showed the greatest 6-min-walk benefit in patients who began the study with the largest functional impairments^91^.

A clinical trial using elamipretide in first time ST-segment elevation myocardial infarction patients did not reduce infarct size^92^. Exclusion of ~40% of the patient population due to patent arteries at the time of reperfusion, and group differences in baseline characteristics may have influenced the results. As we and others have recently noted, a number of factors likely to contribute to lack of translation in cardiac ischemia-reperfusion paradigms^34,93^.

As the sequence of elamipretide contains two non-natural amino acids, there is no known homology between this peptide and endogenous assembly or mitochondrial fusion factors, although the upregulation of cristae assembly factors (such as OPA1) has been recently observed after elamipretide treatment^94,95^. Given the cationic and aromatic repeats, and the fact that many mitochondrial assembly proteins have conserved RYL or RYK motifs^44,96–98^, it is tempting to speculate that the peptide is acting as an enzymatically resistant, cell-permeable analog of an endogenous assembly factor(s). Such a mechanism may explain why there are little/no observable effects in healthy mitochondria across studies with elamipretide as noted above. Healthy inner membranes may be sufficiently intact such that the addition of an adhesion factor has a negligible effect. This type of mechanism may also promote the utility of this peptide (and related analogs) across mitochondrial pathologies that share the commonality of structural abnormalities^3,4^. Clearly further testing is warranted to better elucidate the efficacy of elamipretide across other pathologies.

Our study provides insight into mitochondrial structure-function derangements in acute ischemia-reperfusion by combining functional measurements in mitochondria, fibers, and the intact heart, with new imaging modalities examining cristae architecture. Results indicate that while elamipretide does not prevent the reduction of CL concentration, post-ischemic treatment can improve functional and morphological characteristics of mitochondria even with decreased CL content. These characteristics include improved electron transport chain complex respiration, decreased H_2_O_2_ production, increased mitochondrial density, increased cristae connectivity, enhanced cristae networking within and between mitochondria, and finally, improved inner membrane integrity. The use of biomimetic models has profound potential to expand our understanding of the consequences of phospholipid alterations. Future studies utilizing these approaches will advance the development of mitochondria-targeting strategies.

## Methods

### Animals

Male Sprague–Dawley rats (aged 2–3 months) were used in the study. All procedures received prior approval from the Institutional Animal Care and Use Committees at East Carolina University, Latvian Animal Protection Ethical Committee of Food and Veterinary Service, and Virginia Tech. Animals were housed in a temperature and light-controlled environment and received food and water ad libitum. Prior to excision of the heart, animals received an intraperitoneal (ip) injection of a ketamine/xylazine solution (90 mg/kg/10 mg/kg, respectively), and hearts were excised following the diminution of animal reflexes via midline thoracotomy and placed in ice-cold saline.

### Materials

All phospholipids were purchased from Avanti Polar Lipids Inc. Elamipretide and the TAMRA-elamipretide conjugate were synthesized by New England Peptide. All organic solvents were HPLC grade and all other reagents were purchased from either Fisher Scientific or Sigma.

### I/R and respirometry of permeabilized ventricular fibers

Excised hearts were perfused on one of four modified Langendorff apparatus (AD Instruments) per our established protocols^99,100^. Hearts were exposed to 20/120 min of global ischemia/reperfusion, respectively. For the elamipretide treatment, hearts received 10μM elamipretide beginning at the onset of reperfusion, which is a well-established cardioprotective paradigm in our models^18,78,80,101^. Myocardial oxygen consumption was measured at the end of reperfusion in a subset of hearts per our established protocols^102^. At the end of reperfusion, hearts were split into the experimental groups described below. Two different protocols were employed to determine mitochondrial function after ischemic stress in ventricular muscle fibers. The first set determined mitochondrial function in fibers isolated after cardiac ischemia- reperfusion (“Post I/R Studies”). The second set of studies isolated cardiac fibers from a freshly isolated (normoxic) heart, and then induced anoxia-reoxygenation on the isolated fibers (“A/R Studies”). Detailed methods for the fiber studies are provided in the Methods Supplement. Below (Supplemental Fig. 5) is an overview of the permeabilized experimental flow.

### Isolated mitochondria and BN-PAGE

Mitochondria were isolated from the left ventricle and succinate-derived reverse electron transport determined using our established protocols^103^. Detailed protocols for the separation of native mitochondrial respiratory chain complexes by BN-PAGE are described in the Methods Supplement. Supercomplex flux control coupling factor was measured as described^97^.

### Electron microscopy of mitochondria

A subset of hearts exposed to ischemia-reperfusion (as described above) were utilized for transmission electron microscopy imaging (Virginia Tech Morphology Service Core Laboratory, Virginia-Maryland College of Veterinary Medicine) using modifications to established protocols^104,105^. Serial block-face scanning electron microscopy was done in collaboration with Renovo Neural, Inc. (Cleveland, Ohio). Detailed methods are provided in the Methods Supplement.

### Mass spectrometry

A subset of hearts was taken immediately at the end of reperfusion and the left ventricle was snap frozen, pulverized using a liquid nitrogen-cooled mortar/pestle, and analyzed for CL content and composition using shotgun lipidomics per our established methods^106^. Detailed protocols for the mass spectroscopy studies are provided in the Methods Supplement.

### Construction of biomimetic membranes

A detailed description of the methods for biophysical CL aggregation studies are provided in the Methods Supplement. Pressure-area isotherm experiments were performed using differing biomimetic lipid compositions. For pressure-area isotherms displayed in Fig. 5c, biomimetic mitochondrial lipid monolayers were generated by co-dissolving 40 mol% (18:0-22:6) PC, 30.0 mol% (16:0-20:4) PE, 20 mol% (18:2-18:2-18:2-18:2) CL, 5 mol% (18:1-18:1) PI, 3 mol% (18:1-18:1) PS, and 2 mol% cholesterol in chloroform (10 μg/μL) per our established methods^107^. Pressure-area isotherms displayed in Fig. 5d relied on biomimetic monolayers containing 40 mol% (18:0-22:6) PC, 33.0 mol% (16:0-20:4) PE, 20 mol% (18:2-18:2-18:2-18:2) CL, 5 mol% (18:1-18:1) PS, and 2 mol% cholesterol in chloroform (10 μg/μL). The area per molecule was determined at a physiological surface pressure of 30 mN/m as previously shown^108^. Giant unilamellar vesicles were constructed via electroformation as previously described^30^ and contained 39.9 mol% (18:0-22:6) PC, 30.0 mol% (16:0-20:4) PE, 20 mol% (18:2-18:2-18:2-18:2) CL, 5 mol% (18:1-18:1) PI, 3 mol% (18:1-18:1) PS, 2 mol% cholesterol, and 0.1 mol% NAO. For select experiments, the total CL concentration was decreased 25-50% by mass to reflect changes seen from mass spectroscopy studies. In a subset of studies peptide was added immediately after spotting the lipid monolayer or prior to imaging.

### Statistics and reproducibilty

All data were analyzed using and GraphPad Prism 8 and are presented as mean ± sem. Data were distributed normally, which allowed for parametric analyses. Statistical analyses were conducted using a one-way ANOVA followed by a Bonferroni post-hoc, with *P*-values ≤ 0.05 considered significant. One trace from RET studies was excluded because the hydrogen peroxide emission was greater than two standard deviations away from the mean.

### Reporting summary

Further information on research design is available in the Nature Research Reporting Summary linked to this article.

## Supplementary information

Supplementary Information

Description of Additional Supplementary Files

Supplementary Data 1

Supplementary Data 2

Supplementary Movie 1

Supplementary Movie 2

Supplementary Movie 3

Supplementary Movie 4

Supplementary Movie 5

Supplementary Movie 6

Reporting Summary

## Data Availability

The authors declare that all reported data in the main and supplementary files will be provided to other investigators as requested. Source data underlying plots shown in figures are provided in Supplementary Data 1 and 2.
