# Supplementary material for: The cardiolipin-binding peptide elamipretide mitigates fragmentation of cristae networks following cardiac ischemia reperfusion in rats
Source: Commun Biol. 2020 Jul 17;3:389. doi: 10.1038/s42003-020-1101-3 (PMC7368046; doi:10.1038/s42003-020-1101-3)
Supplement: Supplementary file 1 — Supplementary Information [file 42003_2020_1101_MOESM1_ESM.pdf]

Methods Supplement for Allen & Pennington et al.

Myocardial uptake and mitochondrial localization of elamipretide.

We confirmed that elamipretide localized to the mitochondria in intact, perfused hearts as well as cultured cells. One of the challenges with imaging putative mitochondria-localizing compounds is that fluorescent reporters commonly utilized (BODIPY, rhodamine, thiazole orange) can independently target to mitochondria due to the fluorescent probe's own cationic, lipophilic nature ([1, 2] and DA Brown, unpublished data). Accordingly, we used the fluorophore TAMRA, which is cell permeable but does not localize specifically to mitochondria[3].

Permeabilized ventricular fiber studies.

For <u>Post I/R</u> studies, the left ventricle was dissected after the end of reperfusion and placed on a petri dish containing ice-cold buffer containing in (mM): 7.2 K_2EGTA, 2.8 CaK_2EGTA, 20 Imidazole, 20 Taurine, 5.7 ATP, 14.3 Phosphocreatine, 6.6 $MgCl_2$-$6H_2O$ and 50 MES; pH 7.1. All fat and connective tissue was removed under a dissecting microscope, and small cardiac fiber bundles (5-7 mg of wet weight) were prepared. Fiber bundles were permeabilized using 50μg/ml saponin at 4°C for 20 min. Permeabilized fiber bundles were then washed, 3 times for 5 min each, in ice-cold buffer Z containing in (mM): 110 K-MES, 35 KCL, 1 EGTA, 5 K_2HPO_4, 3 $MgCl_2$-$6H_2O$, and 5 mg/ml BSA; pH 7.4. The fiber bundles were kept at 4°C in buffer Z until analysis.

All experiments were conducted at 37°C in presence of 20 μM blebbistatin to prevent contraction. Five Oroboros High-resolution Respirometers (Oroboros Instruments, Innsbruck, Austria) in the Virginia Tech Metabolism Core were utilized in parallel to measure O_2 consumption and/or H_2O_2 emission per established protocols[4]. Buffer Z was used as respiration medium and O_2 consumption was monitored during a substrate-inhibitor titration ("SUIT") protocol in order to obtain a step-by-step analysis of various components of the

mitochondrial respiratory chain as described by Kuznestov et al.[5]. The addition of substrates and inhibitors was done in the following sequence: glutamate/malate (10mM/5mM), ADP (2 mM), rotenone (0.5μM), succinate (10mM), antimycin A (5μM), N,N,N',N'-tetramethyl-p-phenylenediamine (TMPD)/ascorbate (0.5mM/2mM), cytochrome C (10μM), and FCCP (1μM). At the end of each experiment the fiber bundles used were washed with distilled water and lyophilized in a freeze-dryer (Labconco, Kansas City, MO) for 3 hours and then weighed on a microsclae (Mettler-Toledo XS3DU). O_2 consumption is expressed in pmol/min*mg dry wt. FCCP data were excluded from 3 fibers because they were statistical outliers, with values greater than two standard deviations away from the group mean. Respiratory control ratio in permeabilized fibers was calculated as the ratio of ADP-stimulated respiration divided by respiration with glutamate/malate before the addition of ADP.

For A/R studies in ventricular fibers, mitochondrial respiration and H_2O_2 flux following anoxia-reoxygenation were performed using the Oxygraph-2k platform with established protocols [6-8]. Cardiac fiber bundles were permeabilized using 50μg/ml saponin and 0,5 mg/ml collagenase at 4ºC in 1 ml of buffer A (20mM imidazole, 0.5mM dithiothreitol, 20mM taurine, 7.1mM $MgCl_2$, 50 mM MES, 5 mM ATP, 15 mM phosphocreatine, 2.6 mM CaK_2EGTA, 7.4 mM K_2EGTA, pH 7.0 at 4ºC). After 15 min incubation, fibers were washed for 15 min in 2 ml of buffer B (20mM imidazole, 0.5mM dithiothreitol, 20mM taurine, 1.6mM $MgCl_2$, 100mM MES, 3mM KH_2PO_4, 2.9mM CaK_2EGTA, 7mM K_2EGTA, pH 7.1 at 37ºC) supplemented with elamipretide (100nM) or vehicle. State 3 respiration was stimulated with succinate (10mM), rotenone (0.5μM), and ADP (5mM), and the fiber bundle was left to consume all O_2 in the respiratory chamber (typically within 10-20 min) for the induction of anoxia. After 30 min anoxia, O_2 was reintroduced to the chamber by opening the chamber to achieve reoxygenation. After 8 min of reoxygenation, the chamber was closed and O_2 flux monitored for 2 min. At the end of the experiment antimycin A (2.5μM) was added to determine mitochondrial-independent residual oxygen consumption. H_2O_2 flux was measured with 10μM Amplex Red, 1 U/mL horseradish

peroxidase (HRP) and 5 U/mL superoxide dismutase (SOD), and H_2O_2 was calibrated using serial 0.1 µM H_2O_2 titrations.

Separation of Native Mitochondrial Respiratory Chain Complexes by Blue-Native Polyacrylamide Gel Electrophoresis (BN-PAGE). Separation of mitochondrial respiratory chain complexes was performed using BN-PAGE according to methods described by Schägger et al.[9, 10]. Based on established protocols that differentiate between supercomplexes and individual electron transport chain (ETC) complexes[11, 12], mitochondrial membranes were solubilized using either digitonin (digitonin: mitochondrial protein ratio was 8:1 (w/w)) for isolation of mitochondrial supercomplexes, or DDM (DDM:protein ratio of 2:1) for native complexes I, III, and V in sample buffer. After solubilization, the samples were centrifuged for 30 min at 20,000 x g, and protein content of the supernatant was determined using bicinchoninic acid (BCA protein assay reagent, Thermo Scientific, Rockford, IL, USA). Coomassie G-250 was added to each sample in a ratio detergent-to-dye of 8:1 (w/w), and 35 µg total protein from each sample were separated on a Native PAGE 3-12% Bis-Tris (Life Technologies, Carlsbad, CA, USA) at 4°C. Running buffer was supplemented with 0.02% Coomassie G-250 to allow visualization of protein bands. The stained bands were scanned using the Odyssey Infrared Imaging system (LI-COR Biosciences, Lincoln, NB), and the densitometric analysis was done blinded using Image J software (National Institutes of Health). For densitometric analysis of complex V (Supplemental Figure S2E), all complex V bands were normalized against the sum of each individual band (i.e. complex V band density/(complex I + complex V + complex $(III)_2$ band densities)).

Supercomplex Coupling Control Factor (SCCF). The SCCF has been postulated to reflect the intact nature of supercomplexes, as previously described in failing human heart mitochondria [13]. Using respirometry, we calculated SCCF as the change in respiration with complex I substrates alone (glutamate/malate) divided by the change respiration after succinate addition:

SCCF = [(glutamate+malate) / (glutamate+malate+succinate – (glutamate+malate)], or:

$$\frac{C\ I}{C\ (I + II) - C\ I}$$

Electron microscopy studies.

Transmission Electron Microscopy (TEM). The left ventricle was isolated after I/R and sectioned into 3x3mm pieces. Sectioned tissue was chemically fixed with 3% glutaraldehyde for 24 hours and then washed twice in 0.1M Na-cacodylate for 15 minutes. Following the washes, tissue was post-fixed in 1% osmium tetroxide in 0.1M Na-cacodylate for 1 hour to fix membrane lipids. Samples were dehydrated in graded ethanol solutions of increasing concentrations (15%, 30%, 50%, 70%, 95%, and 100%) for 15 minutes, followed by a 15-minute submersion in propylene oxide. Dehydrated tissue was infiltrated with a 50:50 propylene oxide:Poly/Bed 812 solution for 12 hours. This was followed by a 12-hour infiltration protocol of 100% Poly/Bed 812, so that the sample resin was polymerized into a hard plastic suitable for microtome sectioning. Samples were embedded into molds and cured at 60°C for 48 hours, and then sectioned into 90nm slices using a microtome. Slices were loaded into metal grids and stored in 100% ethanol until ready for use. For imaging, sections were air-dried and then stained with 2% uranyl acetate followed by lead citrate to enhance nucleic acid and cell membrane structure contrasts, respectively. Samples were loaded onto a plate adapter and imaged using transmission electron microscopy (JEOL JEM-1400) with a magnification of 25,000X and resolution of 0.0022um x 0.0022um. Images were processed in Gatan Inc. software and stored on a personal computer. Contact image analysis, sarcomere length measurements, mean mitochondrial Feret diameter [14], cristae complexity index [15], and electron density were done using ImageJ. For about one-half of the images collected, the cristae were not of high enough resolution to accurately measure cristae complexity, but mitochondrial morphology and electron density was still measured for these images. Cristae complexity index is an accepted method [15] in the field

that quantifies mitochondrial cristae integrity. The method quantifies the number of times straight lines spanning across the most medial horizontal and vertical axes of mitochondria intersect with cristae. The method is a simple and time efficient way to quantify the number and integrity of cristae in a given mitochondria. Likewise, not all images that contained mitochondria contained measurable sarcomeres, also reflected by differing n's in the analysis.

Serial Block Face Scanning Electron Microscopy (SBF-SEM). A subset of hearts was fixed with electron microscopy-grade buffer (4% paraformaldehyde, 2.5% glutaraldehyde, 0.2M sodium cacodylate, pH =7). Tissues were stained and imaged by Renovo Neural Inc. (Cleveland, Ohio). Briefly, the samples were washed, stained with 1% tannic acid for 30 mins and then incubated successively with osmium ferrocyanide, thiocarbohydrazide, osmium tetroxide, uranyl acetate and lead aspartate, as previously described[16]. Tissues were dehydrated and embedded in Epon resin (Electron Microscopy Sciences).

Trimmed samples were imaged using either a ThermoFisher VolumeScope system on a Teneo SEM platform, or in a Zeiss Sigma VP scanning electron microscope equipped with a Gatan 3View in-chamber ultramicrotome stage with Gatan low-kV backscattered electron detector. On both systems, stacks of digital images were acquired at 6.0 nm/pixel resolution (x,y) at 2.0 kV, using 65 nm steps (z); these are standard settings that approximate TEM-based imaging. Each resulting serial image stack contained ~500 images (~33µm deep, 49µm high x 49µm wide). Images were scaled and sub-stacks were generated and aligned using Image J software with the FIJI plugin suite. To augment the distinction between the mitochondrial cristae and matrix, slices were contrast enhanced to 0.5%, CLAHE filtered (511 block size, 512 histogram bins), and rendered using a 0.5 gamma processing setting.

For analyses and 3D reconstructions, the 5-10th slices of 8 mitochondria (325-650nm deep) were used across treatment groups. Cristae contact site analyses were performed after contrast enhancement by calculating the percentage of cristae networks adhered to the

intermitochondrial junction on the 5th slice (325nm deep) of all mitochondria across treatments (number of cristae within a cristae network ultimately contacting an intermitochondrial junction / total number of cristae). All cristae longer than 100nm were included in the contact site analyses. A cristae contact site was considered to be present if a cristae longer than 100nm was adhered to a IMJ.

To measure intermitochondrial network connectivity, we analyzed a subset of 8 mitochondrial images per group where 3-7 mitochondria were interconnected with each other. In these images, we utilized the flood-fill macro (ImageJ) to highlight inter-connected contrast. A single flood-fill click on an internal (in between adjacent mitochondria) intermitochondrial junction was performed to initiate intermitochondrial connectivity. The yellow highlighted contrast was subsequently separated from the rest of the network using the color threshold tool (0/38 hue, 0/255 saturation, 0/255 brightness, mean threshold method, black color, HSB color space, dark background). Yellow highlighted networks were then converted to white and the mean grey value was calculated. The percent connectivity was measured by dividing the thresholded mean grey value by the total network's mean grey value (i.e. the grey value before employing flood-fill).

Cristae width was analyzed using a custom Matlab program, previously described [17], modified and combined with a blob algorithm [18] to quantify mitochondrial cristae width. The program calculated, in pixels, the maximal horizontal distance between each side of the cristae. These values were then averaged over the entire array to produce an average mitochondrial cristae width value. The blob algorithm was utilized to weigh the averages by blob size and reduce human intervention in the analysis while quantifying mitochondrial cristae. Furthermore, the blob algorithm was utilized to calculate the total area occupied by networked or orphaned cristae (in pixels).

Lipidomic studies.

Mass spectroscopy for CL analyses. 30 mg of powdered ventricular tissue was used for shotgun lipidomics, with each sample blinded to the lipidomics core. Frozen heart samples were weighed, lyophilized, pulverized, and homogenized in 500 µl of ice-cold diluted phosphate-buffered saline (0.1X PBS) on a cooling tissue homogenizer (Cryolys Precellys Evolution Homogenizer). Protein assays on individual homogenates were performed using a bicinchoninic acid protein assay kit (Pierce, Rockford, IL, USA). Lipids were extracted by a modified procedure of Bligh and Dyer extraction as described previously[19, 20] in the presence of an internal standard (14:1-14:1-14:1-14:1 CL, 2 nmol/mg protein) which were added based on total protein content of the sample. Each lipid extract was resuspended into a volume of 200µL of chloroform/methanol (1:1, v/v) per mg of protein and flushed with nitrogen, capped, and stored at −20 °C for lipid analysis. For electrospray ionization (ESI) direct infusion analysis, lipid extract was further diluted to a final concentration of ~500 fmol/µL by $CHCl_3$/MeOH/isopropanol (1/2/4, v/v/v), and the mass spectrometric analysis was performed on a Q-Exactive mass spectrometer (Thermo Scientific, San Jose, CA) equipped with an automated nanospray device (TriVersa NanoMate, Advion Bioscience Ltd., Ithaca, NY) and operated with Xcalibur software. Identification and quantification of CL molecular species were performed using an automated software program [21]. The determined CL levels were normalized to the protein content of individual samples.

Biophysical membrane studies.

All phospholipids were handled with extreme care to prevent oxidation under low light conditions and a gentle stream of nitrogen gas using established protocols. The levels of PC, PE, CL, PI, PS, and cholesterol modeled the mitochondrial inner membrane phospholipidome and were consistent with the literature [22].

Construction of monolayers for pressure-area studies. Pressure-area isotherms were generated using a Mini Langmuir-Blodgett Trough (KSV NIMA, Biolin Scientific, Paramus, NJ) as previously demonstrated [23]. Fresh lipid stocks in chloroform (HPLC grade, Fisher Scientific) were used for all studies. Prior to collecting pressure-area isotherms the trough was washed three times with 70% ethanol, Milli-Q water, and subphase. Lipid monolayers were constructed by spotting 10 µg of lipid on a subphase of 10 mM sodium phosphate buffer (pH 7.4). The addition of peptide (1 uM final concentration) occurred immediately after spotting the lipid film. In a subset of studies (Figure 5E) CL was oxidized by >24h of exposure to room air prior to lipid monolayer formation, a paradigm that is established to evoke CL peroxidation [24].

Construction of biomimetic Giant Unilamellar Vesicles (GUVs) for imaging microdomains. Electroformation occurred at room temperature using a buffer containing 10 mM HEPES (pH 7.4) and 250 mM sucrose as previously described [25]. For select experiments, peptide (20 uM final concentration) was immediately added to the vesicles prior to being drawn into a glass rectangular micro-capillary tube and mounted onto a microscope slide. Samples were imaged at room temperature (23 °C).

CL lipid vesicle aggregation assays. Lipid vesicles were prepared as previously described [26]. Briefly, lipids (either pure PC (control) or pure CL) (1mg total) were dissolved in HPLC-grade chloroform, extensively dried briefly under a gentle N_2 stream and was immediately followed by vacuum drying for 1 hr. The lipid film was hydrated in 1 ml of 80 mM sodium phosphate buffer (pH = 7.4) and occurred above the highest phase transition temperature (T_m). The aqueous dispersions were subjected to three freeze-thaw cycles using liquid N_2 and a warm water bath. CL-enriched vesicles precipitate out of solution upon the addition of elamipretide, we monitored the optical density at 300 nm to quantify the extent of CL-elamipretide aggregation.

References

1. Smith, R.A., R.C. Hartley, and M.P. Murphy, *Mitochondria-targeted small molecule therapeutics and probes.* Antioxid Redox Signal, 2011. **15**(12): p. 3021-38.
2. Yousif, L.F., et al., *Mitochondria-penetrating peptides: sequence effects and model cargo transport.* Chembiochem, 2009. **10**(12): p. 2081-8.
3. Alta, R.Y., et al., *Mitochondria-penetrating peptides conjugated to desferrioxamine as chelators for mitochondrial labile iron.* PLoS One, 2017. **12**(2): p. e0171729.
4. Perry, C.G., et al., *Inhibiting myosin-ATPase reveals a dynamic range of mitochondrial respiratory control in skeletal muscle.* Biochem J, 2011. **437**(2): p. 215-22.
5. Kuznetsov, A.V., et al., *Analysis of mitochondrial function in situ in permeabilized muscle fibers, tissues and cells.* Nat Protoc, 2008. **3**(6): p. 965-76.
6. Makrecka-Kuka, M., G. Krumschnabel, and E. Gnaiger, *High-Resolution Respirometry for Simultaneous Measurement of Oxygen and Hydrogen Peroxide Fluxes in Permeabilized Cells, Tissue Homogenate and Isolated Mitochondria.* Biomolecules, 2015. **5**(3): p. 1319-38.
7. Kuka, J., et al., *The cardioprotective effect of mildronate is diminished after co-treatment with L-carnitine.* J Cardiovasc Pharmacol Ther, 2012. **17**(2): p. 215-22.
8. Makrecka, M., et al., *Mildronate, the inhibitor of L-carnitine transport, induces brain mitochondrial uncoupling and protects against anoxia-reoxygenation.* Eur J Pharmacol, 2014. **723**: p. 55-61.
9. Schagger, H., *Respiratory chain supercomplexes of mitochondria and bacteria.* Biochim Biophys Acta, 2002. **1555**(1-3): p. 154-9.
10. Schagger, H., W.A. Cramer, and G. von Jagow, *Analysis of molecular masses and oligomeric states of protein complexes by blue native electrophoresis and isolation of membrane protein complexes by two-dimensional native electrophoresis.* Anal Biochem, 1994. **217**(2): p. 220-30.
11. Gomez, L.A., et al., *Supercomplexes of the mitochondrial electron transport chain decline in the aging rat heart.* Arch Biochem Biophys, 2009. **490**(1): p. 30-5.
12. Wittig, I., H.P. Braun, and H. Schagger, *Blue native PAGE.* Nat Protoc, 2006. **1**(1): p. 418-28.
13. Chatfield KC, S.G., Chau S, Phillips EK, Ambardekar AV, Aftab M, Mitchell MB, Sucharov CC, Miyamoto SD, Stauffer BL, *Elamipretide Improves Mitochondrial Function in the Failing Human Hear.* JACC Basic Translational Science 2019. **4**(2): p. 147-157.
14. Federico, M., et al., *Calcium-calmodulin-dependent protein kinase mediates the intracellular signalling pathways of cardiac apoptosis in mice with impaired glucose tolerance.* J Physiol, 2017. **595**(12): p. 4089-4108.
15. Williams, P.A., et al., *Retinal ganglion cell dendritic degeneration in a mouse model of Alzheimer's disease.* Neurobiol Aging, 2013. **34**(7): p. 1799-806.
16. Mukherjee, K., et al., *Analysis of Brain Mitochondria Using Serial Block-Face Scanning Electron Microscopy.* J Vis Exp, 2016(113).
17. Raisch, T., M. Khan, and S. Poelzing, *Quantifying Intermembrane Distances with Serial Image Dilations.* Journal of Visualized Experiments, 2018. **139**.
18. Analyst, I., *Image Segmentation Tutorial.* 2015.

19. Han, X., *Lipidomics: Comprehensive Mass Spectrometry of Lipids*. 2016, Hoboken, New Jersey: John Wiley & Sons, Inc. 496.
20. Han, X., *Comprehensive Mass Spectrometry of Lipids*. 2016: John Wiley & Sons, Inc.
21. Yang, K., et al., *Automated lipid identification and quantification by multi-dimensional mass spectrometry-based shotgun lipidomics.* Anal. Chem., 2009. **81**: p. 4356-4368.
22. Osman, C., D.R. Voelker, and T. Langer, *Making heads or tails of phospholipids in mitochondria.* J Cell Biol, 2011. **192**(1): p. 7-16.
23. Pennington, E.R., et al., *Distinct membrane properties are differentially influenced by cardiolipin content and acyl chain composition in biomimetic membranes.* Biochim Biophys Acta, 2017. **1859**(2): p. 257-267.
24. Kim, J., et al., *Cardiolipin: characterization of distinct oxidized molecular species.* J Lipid Res, 2011. **52**(1): p. 125-35.
25. Pennington ER, S.E., Fix A, Dadoo S, Zeczycki TN, DeSantis A, Schlattner U, Coleman R, Chicco AJ, Brown DA, Shaikh SR, *Proteolipid domains form in biomimetic and cardiac mitochondrial vesicles and are regulated by cardiolipin concentration but not monolyso-cardiolipin.* Journal of Biological Chemistry, 2018. **293**(41): p. 15933-15946.
26. Shaikh, S.R., et al., *Oleic- and docosahexaenoic acid-containing phosphatidylethanolamines differentially phase separate from sphingomyelin.* Biochim Biophys Acta, 2009. **1788**(11): p. 2421-6.

Supplemental Figures for Allen & Pennington et al.

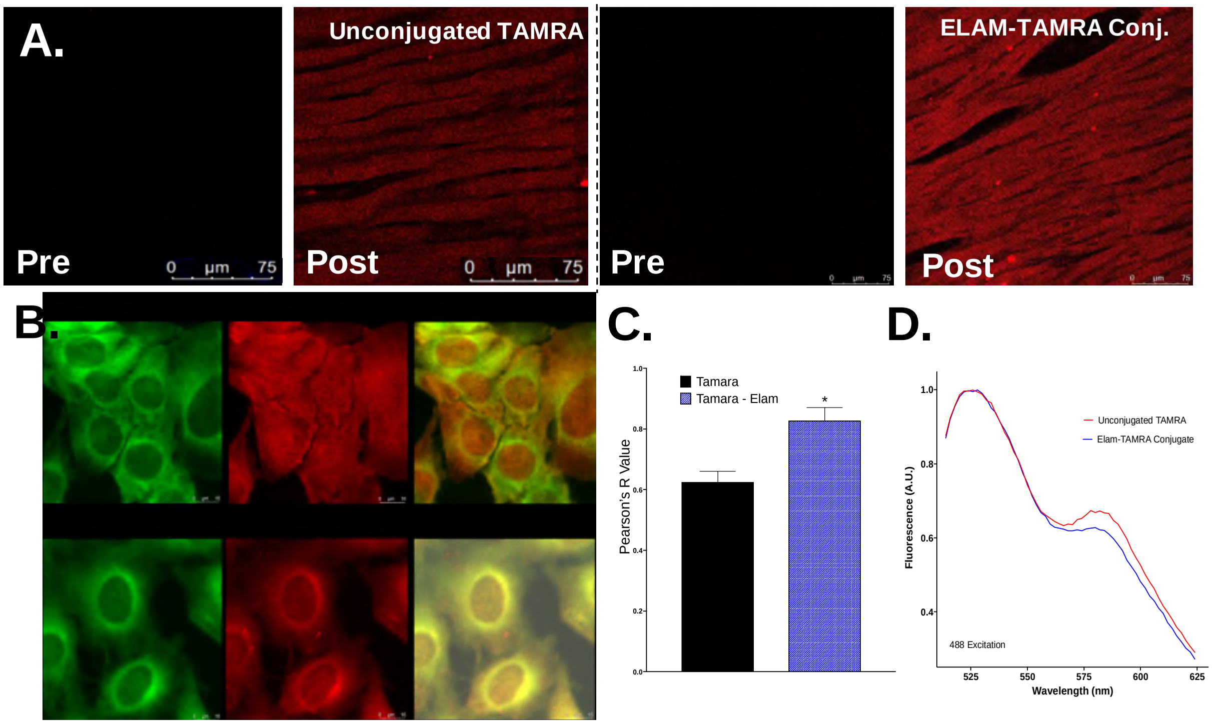

Supplemental Figure 1.

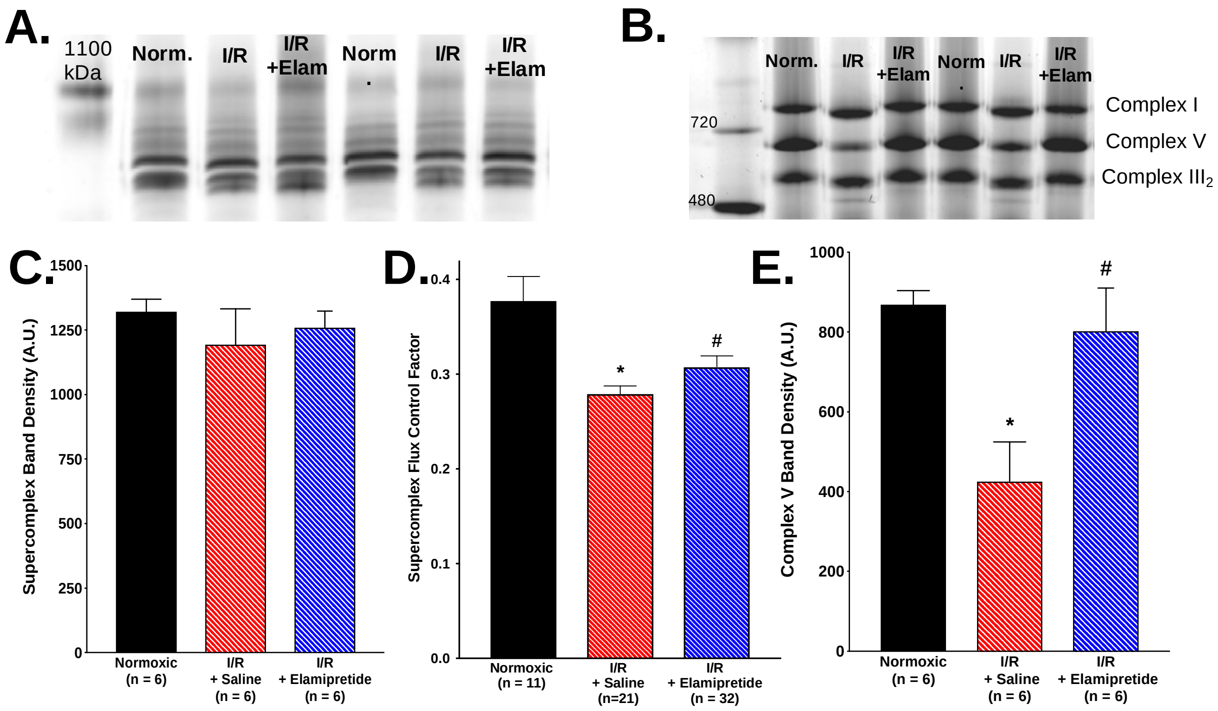

Supplemental Figure 2.

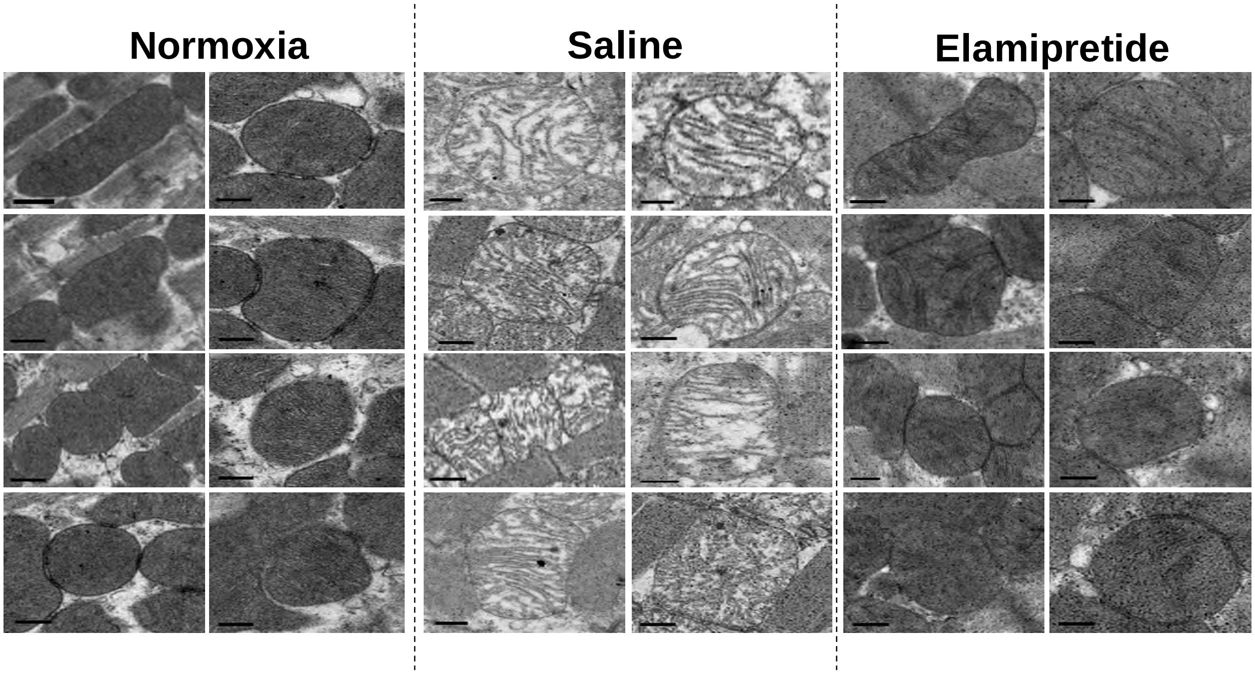

Supplemental Figure 3.

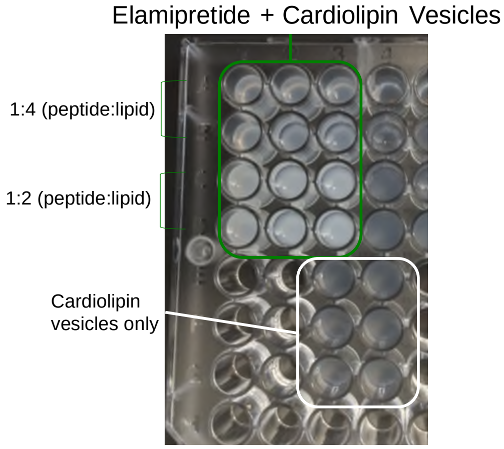

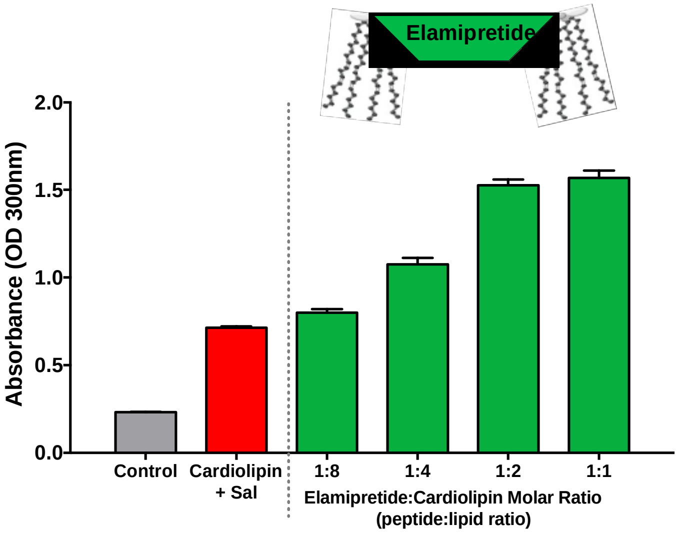

Supplemental Figure 4.
