# Supplementary material for: The cardiolipin-binding peptide elamipretide mitigates fragmentation of cristae networks following cardiac ischemia reperfusion in rats
Source: Commun Biol. 2020 Jul 17;3:389. doi: 10.1038/s42003-020-1101-3 (PMC7368046; doi:10.1038/s42003-020-1101-3)
Supplement: Supplementary file 2 — Description of Additional Supplementary Files [file 42003_2020_1101_MOESM2_ESM.pdf]

Description of Additional Supplementary Files

File Name: **Supplementary Data 1**
Description: Dataset 1 capture all raw data used for Figure 1

File Name: **Supplementary Data 2**
Description: Dataset 2 capture all raw data used for Figure 2-5 as well as Supplemental Figures 1-3 and 10

File Name: **Supplementary Movie 1**
Description: Representative SBF-SEM movie of mitochondria from normoxic hearts

File Name: **Supplementary Movie 2**
Description: Representative SBF-SEM movie of mitochondria from I/R + saline hearts

File Name: **Supplementary Movie 3**
Description: Representative SBF-SEM movie of mitochondria from I/R + Elam. hearts

File Name: **Supplementary Movie 4**
Description: Representative SBF-SEM movie of cristae networking within one cross-sectioned normoxic mitochondrion. Yellow cristae are networked while red cristae are orphaned

File Name: **Supplementary Movie 5**
Description: Representative SBF-SEM movie of cristae networking within one cross-sectioned I/R + saline mitochondrion. Yellow cristae are networked while red cristae are orphaned

File Name: **Supplementary Movie 6**
Description: Representative SBF-SEM movie of cristae networking within one cross-sectioned I/R + Elam. mitochondrion. Yellow cristae are networked while red cristae are orphaned
