# Supplementary material for: The cardiolipin-binding peptide elamipretide mitigates fragmentation of cristae networks following cardiac ischemia reperfusion in rats
Source: Commun Biol. 2020 Jul 17;3:389. doi: 10.1038/s42003-020-1101-3 (PMC7368046; doi:10.1038/s42003-020-1101-3)
Supplement: Supplementary file 11 — Reporting Summary [file 42003_2020_1101_MOESM11_ESM.pdf]

nature research

Corresponding author(s): Saama Raza Shaikh
shaikhsa@email.unc.edu

Last updated by author(s): Jun 11, 2020

Reporting Summary

Nature Research wishes to improve the reproducibility of the work that we publish. This form provides structure for consistency and transparency in reporting. For further information on Nature Research policies, see Authors & Referees and the Editorial Policy Checklist.

Statistics

For all statistical analyses, confirm that the following items are present in the figure legend, table legend, main text, or Methods section.

n/a	Confirmed	
☐	☒	The exact sample size (n) for each experimental group/condition, given as a discrete number and unit of measurement
☐	☒	A statement on whether measurements were taken from distinct samples or whether the same sample was measured repeatedly
☐	☒	The statistical test(s) used AND whether they are one- or two-sided<br>*Only common tests should be described solely by name; describe more complex techniques in the Methods section.*
☒	☐	A description of all covariates tested
☐	☒	A description of any assumptions or corrections, such as tests of normality and adjustment for multiple comparisons
☐	☒	A full description of the statistical parameters including central tendency (e.g. means) or other basic estimates (e.g. regression coefficient) AND variation (e.g. standard deviation) or associated estimates of uncertainty (e.g. confidence intervals)
☒	☐	For null hypothesis testing, the test statistic (e.g. F, t, r) with confidence intervals, effect sizes, degrees of freedom and P value noted<br>*Give P values as exact values whenever suitable.*
☒	☐	For Bayesian analysis, information on the choice of priors and Markov chain Monte Carlo settings
☒	☐	For hierarchical and complex designs, identification of the appropriate level for tests and full reporting of outcomes
☒	☐	Estimates of effect sizes (e.g. Cohen's d, Pearson's r), indicating how they were calculated

Our web collection on statistics for biologists contains articles on many of the points above.

Software and code

Policy information about availability of computer code

Data collection	N/A
Data analysis	Graph Pad Prism v7; NIH ImageJ was used for image analyses.

For manuscripts utilizing custom algorithms or software that are central to the research but not yet described in published literature, software must be made available to editors/reviewers. We strongly encourage code deposition in a community repository (e.g. GitHub). See the Nature Research guidelines for submitting code & software for further information.

Data

Policy information about availability of data

All manuscripts must include a data availability statement. This statement should provide the following information, where applicable:

- Accession codes, unique identifiers, or web links for publicly available datasets
- A list of figures that have associated raw data
- A description of any restrictions on data availability

All raw data are available upon request.

Field-specific reporting

Please select the one below that is the best fit for your research. If you are not sure, read the appropriate sections before making your selection.

☒ Life sciences ☐ Behavioural & social sciences ☐ Ecological, evolutionary & environmental sciences

For a reference copy of the document with all sections, see nature.com/documents/nr-reporting-summary-flat.pdf

Life sciences study design

All studies must disclose on these points even when the disclosure is negative.

Sample size	No power analyses was performed. Sample sizes were chosen based on previous experiments and/or previously published protocols. For SBF-SEM studies one animal heart was used per treatment group, eight individual mitochondria were chosen for analyses. Sample size limitations were restricted due to analysis time and cost variables. SBF-SEM studies were complimented with higher sample sizes for TEM studies.
Data exclusions	No data were excluded as indicated.
Replication	All animal studies were performed on separate days using different animals from the same strain. Mitochondria was isolated fresh, immediately after harvesting animal hearts. For biophysical studies, experiments were performed on separate days using different lipid stocks to ensure reproducibility.
Randomization	Animals were randomly grouped. All animals were under the same dietary restrictions, standard rodent chow.
Blinding	Biophysical imaging analyses and western blot analyses were blinded. TEM and SBF-SEM sample processing was blinded.

Reporting for specific materials, systems and methods

We require information from authors about some types of materials, experimental systems and methods used in many studies. Here, indicate whether each material, system or method listed is relevant to your study. If you are not sure if a list item applies to your research, read the appropriate section before selecting a response.

Materials & experimental systems

n/a	Involved in the study	
☒	☐	Antibodies
☒	☐	Eukaryotic cell lines
☒	☐	Palaeontology
☐	☒	Animals and other organisms
☒	☐	Human research participants
☒	☐	Clinical data

Methods

n/a	Involved in the study	
☒	☐	ChIP-seq
☒	☐	Flow cytometry
☒	☐	MRI-based neuroimaging

Animals and other organisms

Policy information about studies involving animals; ARRIVE guidelines recommended for reporting animal research

Laboratory animals	Male Sprauge-Dawley rats were used at an age of 2-3 months. All animals were fed a standard rodent chow. All animals were housed in temperature and light controlled environments.
Wild animals	N/A
Field-collected samples	N/A
Ethics oversight	Approved by Animal Use Committee

Note that full information on the approval of the study protocol must also be provided in the manuscript.
